# Report of Rochester Hahnemannian Society

**Published:** 1889-02

**Authors:** W. H. Baker

**Affiliations:** Secretary


### REPORT OF ROCHESTER HAHNEMANNIAN SOCIETY.

The regular monthly meeting of the Rochester Hahnemannian Society was held at the office of Dr. Biegler, November 20th, 1888, President R. C. Grant in the chair.

Members present: Drs. Grant, Biegler, Schmitt, Brownell, Johnson, Hoard, Hermance, Baker, Carr.

Minutes of last meeting read and approved.

Sections 169 to 180 of the *Organon* were read, with the following discussion:

Dr. Johnson—These sections explain the sections read at last meeting, also give explanation of the case that Dr. Schmitt reported.

Dr. Schmitt—All of us have had cases where symptoms have been developed by a remedy, and leading us to the curative drug.

Dr. Brownell—I think the first section read a little confusing. It speaks of a remedy being homœopathic to one portion of the symptoms, and another remedy homœopathic to the other portion. As I understand it, we can only have one homœopathic remedy—the simillimum.

Dr. Schmitt—I think that the point Hahnemann wants to make is, that if you have two remedies that are seemingly indicated, one remedy covering a portion of the symptoms, and a second remedy covering the remaining portion, you are not to give the second remedy after the first before you have examined the case again.

Dr. Grant—It is customary with me to make a note of a remedy to see next, but I seldom select that remedy. A second examination generally brings out a different drug.

Dr. Biegler—That has been a practice with me for a long time, and my experience is the same. I seldom select the remedy that I note to see next.

Dr. Johnson—I would like to ask Dr. Brownell the result of the use of Plantago in the case reported last meeting?

Dr. Brownell—It did not affect the case any; further inquiry developed a history of suppressed foot-sweat, so I gave Silicea, which caused a partial recurrence of the foot-sweat. There is no sugar in the urine now, but he is not well; at present he is on Chelidon. I would like to ask Dr. Biegler if he uses a knife in the treatment of a carbuncle?

Dr. Biegler—I do not; it is bad treatment; with the indicated remedy you will do more for your patient, quiet the pain attending the disease, and make him comfortable. I believe the use of the knife will make matters worse.

Dr. Carr—I have now a case of carbuncle under treatment. It first appeared as one large pimple surrounded by a number of smaller ones, that finally coalesced into one; the opening was as large as a half-dollar. I gave first Hepar-sulph., followed by Lachesis, the color having changed to a purple, with great pain. I have found the yeast poultice one of the best dressings for diseases of this kind—it allays the irritation and has a soothing effect, which is very gatifying to the patient. I look to my remedy for relieving the pain, and Lachesis kept this case comfortable.

The poultice is made from one teacupful of bran, one tablespoonful of flour; add water to make a paste, then add two teaspoonfuls of brewer's yeast (a yeast cake may be used), place in a linen bag and apply; it should be changed about every eight hours.

Dr. Schmitt—I was taught to cut a carbuncle and apply caustic. The first case I treated homœopathically was with six doses of $Sulph^{30}$, and the yeast poultice.

Dr. Brownell reported sequel to case of diphtheria reported by him at the July meeting, and published in the October number of THE HOMŒOPATHIC PHYSICIAN.

### SEQUEL TO A CASE OF DIPHTHERIA TREATED BY LAC-CANINUM.

Tommie H——. About three weeks succeeding the manifestation of the diphtheritic disease in the case reported to this Society in which $Lac\text{-}caninum^{cm}$ proved curative, I was consulted for a condition of paralysis of the muscles of the neck, which has become quite marked, so much as to cause a falling forwards of the head so that it rests on the upper portion of the sternum. There is return of fluids through the nose, and an evident weakness of the muscles of the upper part of the back. Phos. on general principles.

August 6th.—His father brings the boy back in a much worse condition than before, the paralysis being more pronounced, and some staggering in his walk. Complains of stiffness and soreness of the muscles of the neck, on which I prescribed Rhus-tox.$^{cm}$.

August 16th—The family has become anxious at the constantly increasing paralysis, and insists that something further be done, and asked in regard to electricity. I advised one more trial and gave Lac-can.$^{cm}$, of which he received one dose, which restored the use of the muscles, and the boy remains well.

Dr. Carr was appointed essayist for the next meeting. Adjourned to the office of Dr. Schmitt in one month.

W. H. BAKER, *Secretary.*

